# Crystals in rhyolitic melts can nucleate multiple bubbles: an experimentally constrained model

**DOI:** 10.1007/s00445-026-02048-7

**Published:** 2026-09-29

**Authors:** Quinn Johnson, James E. Gardner, Wade Aubin, Patrick Sullivan, Fabian B. Wadsworth, Edward W. Llewellin, Tamara Carley, Halim Kusumaatmaja, Dork Sahagian

**Affiliations:** 1 Department of Earth and Planetary Sciences, Jackson School of Geosciences, University of Texas at Austin, Austin, TX 78712 USA; 2 Department of Physical and Environmental Sciences, Colorado Mesa University, Grand Junction, CO 81501 USA; 3 Earth Sciences, Durham University, Durham, DH1 3LE UK; 4 Earth & Environmental Sciences, Ludwig-Maximilians-Universität-München, 80333 Munich, Germany; 5 Department of Geology and Environmental Geosciences, Lafayette College, Easton, PA USA; 6 Institute for Multiscale Thermofluids, School of Engineering, The University of Edinburgh, Edinburgh, EH9 3FB UK; 7 Department of Earth and Environmental Sciences, Lehigh University, Bethlehem, PA USA

**Keywords:** Heterogeneous bubble nucleation, Nucleation theory, Magma degassing, Rhyolite, Volcanic eruption

## Abstract

Heterogeneous nucleation of bubbles on crystals in magma is thought to be an important driver of volcanic eruptions. Decompression experiments were carried out to elucidate the kinetics of H_2_O bubble nucleation on crystals of different sizes. Samples used were mixtures of hydrated rhyolitic melt and populations of augite or hornblende crystals of < 20 µm or 20–40 µm. We found that bubbles nucleated on surfaces of both types of crystals at supersaturation pressures ≤ 10 MPa, which is a small fraction of the supersaturation needed to homogeneously nucleate H_2_O bubbles in the melt. Very small crystals (< 5–10 µm) will nucleate only ~ 1 bubble, whereas on larger crystals, the number of bubbles that nucleate at a given supersaturation increases with an increased crystal surface area. In addition, the number of bubbles that nucleate on crystals of a given surface area increases with increasing supersaturation, indicating that the distance between nucleation sites decreases with increased supersaturation. We propose that the closer spacing of nucleation sites at higher supersaturation arises from the competition between bubble nucleation rate and the rate of H_2_O diffusion. Based on these results, we develop a modified heterogeneous nucleation rate equation that accounts for the control of crystal surface area on heterogeneous bubble nucleation and the resulting bubble number density generated. We provide a spreadsheet that allows users to calculate both total bubble number density and number densities per crystal size for a specified crystal-size population and different conditions (H_2_O content, temperature, and final pressure).

**Supplementary information:**

The online version contains supplementary material available at https://doi.org/10.1007/s00445-026-02048-7.

## Introduction

Bubbles drive volcanic eruptions, and it is bubble-scale processes that underpin explosive eruptive behavior (Verhoogen 1951; Sparks 1978). Determining the rates at which bubbles nucleate is thus critical for understanding volcanism on Earth and other planets. Gas bubbles can nucleate either homogeneously within pure liquid or heterogeneously, where bubbles form on substrates such as crystals in magma (Toramaru 1995; Mourtada-Bonnefoi and Laporte 1999, 2002; Gardner et al. 1999; Mangan and Sisson 2000; Iacono-Marziano et al. 2007; Gardner 2007; Larsen 2007; Cluzel et al. 2008; Cichy et al. 2011; Gardner 2012; Fiege and Cichy 2015; Le Gall and Pichavant 2016; Shea 2017; Plese et al. 2018; Cáceres et al. 2020; Gardner et al. 2023). Gardner et al. (2023) proposed that during magma ascent, conditions may lead to bubbles nucleating via both mechanisms, leading to an overall system-scale bubble nucleation rate J, which is the sum of the heterogeneous and homogeneous contributions, ${J}_{\mathrm{H}\mathrm{O}\mathrm{M}}$ and ${J}_{\mathrm{H}\mathrm{E}\mathrm{T}}$:

1 $$\begin{array}{l}J=J_{HOM}+J_{HET}=J_oexp\left(-\frac{16\pi\sigma^3}{3k_BT\triangle p^2}\right)\\+\Sigma\beta_iJ_iexp\left(-\frac{16\pi\sigma^3}{3k_BT\triangle p^2}\alpha_i\right)\end{array}$$

where ${J}_{o}$ is the homogeneous nucleation prefactor, ${J}_{i}$ is the pre-factor for the *i*th heterogeneous site (e.g., the *i*th crystal phase), σ is surface tension, ${k}_{B}$ is Boltzmann’s constant, T is temperature, Δp is the difference between the saturation pressure and the melt pressure, and ${\alpha}_{i}$ is the heterogeneous adjustment factor for the *i*th phase (see Table 1 for complete list of variables used in this study). The parameter ${\beta}_{i}$ was added to account for the number density of *i*th sites.

**Table 1:** Notations used in the text

Term	Definition
J (m^−3^ s^−1^)	Overall system-scale bubble nucleation rate
${J}_{\mathrm{H}\mathrm{E}\mathrm{T}}$ (m^−3^ s^−1^)	Heterogeneous contribution to J
${J}_{\mathrm{H}\mathrm{O}\mathrm{M}}$ (m^−3^ s^−1^)	Homogeneous contribution to J
${J}_{0}$ (m^−3^ s^−1^)	Homogeneous nucleation pre-factor
${J}_{i}$ (m^−3^ s^−1^)	Heterogeneous nucleation pre-factor for the *i*th phase
σ (N m^−1^)	Surface tension
${k}_{B}$ (J K^−1^)	Botzmann’s constant
T (K)	Temperature
Δp (MPa)	Difference between the saturation and melt pressures
${\alpha}_{i}$	Heterogeneous adjustment factor for the *i*th phase (also defined as ${\alpha}_{i}={\left({\Delta p}_{\mathrm{H}\mathrm{O}\mathrm{M}}/{\Delta p}_{\mathrm{H}\mathrm{E}\mathrm{T}}\right)}^{2}$ in Eq. 2)
${\beta}_{i}$	Empirical shape/size factor for the *i*th phase proposed by Gardner et al. (2023)
${\Delta p}_{\mathrm{H}\mathrm{O}\mathrm{M}}$ (MPa)	Minimum supersaturation for homogeneous nucleation
${\Delta p}_{\mathrm{H}\mathrm{E}\mathrm{T}}$ (MPa)	Minimum supersaturation for heterogeneous nucleation
${t}_{nuc}$ (s)	Average time for a bubble to nucleate on a given surface
${J}^{\prime}_{\mathrm{H}\mathrm{E}\mathrm{T}}$ (m^−2^ s^−1^)	Number of bubbles per unit area per unit time
${A}_{nuc}$ (m^−2^)	Area of the crystal per bubble
l (m)	Characteristic diffusion length for degassing into bubble
*D* (m^2^ s^−1^)	H_2_O diffusivity in silicate melt
${N}^{\prime}$ (m^−2^)	Number of bubbles per area of crystal
${J}_{c}$ (m^−2^ s^−1^)	Heterogeneous nucleation pre-factor used as a fitting parameter
χ	Correction function for small crystals
A (m^2^)	Total area of crystal
${N}_{b}$ (m^−3^)	Number density of bubbles nucleated in magmas containing crystals
${N}_{c}$ (m^−3^)	Crystal number density
F(A)	Crystal area surface area distribution as a function of crystal area

Whereas homogeneous nucleation is reasonably well understood, with extensions beyond classical nucleation theory able to predict ${J}_{\mathrm{H}\mathrm{O}\mathrm{M}}$ for rhyolitic magmas to reasonable accuracy (e.g., Gonnermann and Gardner 2013; Hajimirza et al. 2019; 2021), ${J}_{\mathrm{H}\mathrm{E}\mathrm{T}}$ is less well-constrained. Importantly, the efficiency of heterogeneous nucleation depends on the type of crystal present as expressed through ${\alpha}_{i}$, which has been approximated for only magnetite, hematite, clinopyroxene, and plagioclase (Hurwitz and Navon 1994; Gardner and Denis 2004; Larsen 2007). Numbers, sizes, and shapes of crystals should also impact heterogeneous nucleation (Schmelzer 1986; Schmelzer and Mahnke 1986; Baidakov and Boltachev 1999; Qian and Ma 2009; Hedges and Whitelam 2012), all of which are implicitly included in ${\beta}_{i}$, but neither ${\beta}_{i}$ nor ${J}_{i}$ have been calibrated, tested, or derived.

In this study, we perform decompression experiments targeted to calibrate ${\alpha}_{i}$ for clinopyroxene and hornblende, both common minerals in rhyolite magma. We find that both are very efficient sites for nucleating H_2_O bubbles, allowing nucleation at supersaturations (Δp) as low as 5 MPa. We also find that the number of bubbles nucleated on crystals larger than ~5 µm is controlled by the available surface area and Δp. Based on these results, we modify the formulation of the heterogeneous term in Eq. 1 by combining ${\beta}_{i}$ and ${J}_{i}$ into a single heterogeneous rate term, ${J}_{\mathrm{c}}$, that captures the microphysics of heterogeneous nucleation, aligning better with nucleation theory (e.g., Blander and Katz 1975).

## Methods

Experiments consisted of mixtures of hydrated silicate glass and crystals. The glass was made using 3-mm-diameter cores drilled from a high-silica rhyolitic obsidian (Table 2). Each core, and enough distilled H_2_O to saturate it at experimental run conditions, was placed inside a gold capsule that had been welded shut at one end. The other end was then welded, and the capsule was heated to 110 °C for 5 min to ensure there were no leaks in the welds. A sealed capsule and Ni filler rod were placed inside a cold-seal pressure vessel, which was then pressurized by water to 150 MPa and heated to 850 °C for 5 days (time sufficient for diffusive hydration of the core; Gardner et al. 2019). The hydrated rhyolite glass was then crushed and ground to < 45 µm with an agate mortar and pestle.

**Table 2:** Compositions of starting materials

	Glass	Augite	Hornblende
SiO_2_	76.53	56.11(89)	52.01(42)
TiO_2_	0.06	0.06(9)	1.34(8)
Al_2_O_3_	13.01	1.87(105)	5.12(18)
FeO*	0.79	6.82(38)	7.77(33)
MnO	0.08	0.16(3)	0.03(2)
MgO	0.02	20.32(38)	18.81(14)
CaO	0.74	12.76(33)	10.64(8)
Na_2_O	3.87	0.41(30)	3.00(8)
K_2_O	4.91	0.15(12)	0.27(2)

All oxides in wt.%; total iron reported as FeO. Mineral compositions from this study, measured using an electron microprobe. Glass composition from Gonnermann and Gardner (2013)

Crystals used were either augite (NMNH R3035) or hornblende (NMNH 117329-1) obtained from the Smithsonian Institution. Mineral compositions are given in Table 2. Each crystal was crushed in a steel mortar, and the fragments were wet sieved to be between 4.5 ϕ and 5.5 ϕ, referred to as the 20–45 µm size fraction. A separate sample of < 5.5 ϕ fragments (≤ 20 µm) was also used.

An experiment consisted of ~40 mg of hydrated glass powder mixed with ~ 0.6 (± 0.1) mg of crystals, which approximates 1 vol% crystal contents. The mixture was poured into a 4-mm-diameter gold capsule that had been welded shut at one end. The other end was left open so that the sample could equilibrate with the pressurizing H_2_O to facilitate sintering of the particles (Gardner et al. 2018, 2019; Aubin and Gardner 2024). The capsule was placed inside the sample cup at one end of an Inconel rod, which was then inserted into a pressure vessel equipped with a rapid quench extension (Gardner 2009). The sample was held in the water-cooled chamber of the vessel while the pressure vessel equilibrated to experimental conditions of 875 °C and 150 MPa. Once equilibrated, the sample was raised into the hot zone using an external magnet and held at those conditions for 1 h while the mixture sintered. After 1 h, pressure was increased rapidly to 200 MPa and held for 10 min in order to resorb as many bubbles trapped during sintering as possible (Gardner et al. 2018, 2019). The higher pressure raises the solubility of H_2_O in the melt resulting in some H_2_O held in bubbles formed by sintering to dissolve into the melt and thereby shrinking (or completely resorbing) sintering bubbles. After 10 min, there are far fewer bubbles still present in the samples than before compression. Seven samples were quenched after the 10 min by rapidly lowering them back into the water-cooled jacket, where they cooled at ~150 °C per second (Gardner et al. 2018). Eight other samples were decompressed to pressures between 150 and 185 MPa in ~ 1–2 s and held at the new lower pressure for 5 min before being quenched. Note that part of one sample (Q-90) that had been quenched at 200 MPa was used as starting material for one decompression experiment (Q-108) that followed all other protocols.

All samples were prepared as thick sections for analysis. The seven samples quenched at 200 MPa were doubly polished and analyzed for dissolved H_2_O contents using Fourier Transform Infrared (FTIR) spectroscopy (Table 3). H_2_O contents were determined from measured peak absorbances at 5250 cm^−1^ and 4500 cm^−1^ wavenumbers using the model of Zhang et al (1997). Sample thickness was measured at each analyzed spot by focusing through the sample using a petrographic microscope that is equipped with a Heidenhain linear encoder that measures the vertical motion of the microscope stage (Gardner et al. 2013; Aubin and Gardner 2024). H_2_O contents listed in Table 3 are averages of 4–8 measurements, and uncertainties are the standard error on those averages.

**Table 3:** Sintering and compression experiments

Run	Crystal^a^	T^b^ (°C)	P_1_^c^ (MPa)	Time_1_^c^ (min)	P_2_^c^ (MPa)	Time_2_^c^ (min)	H_2_O^d^ (wt%)	Bubbles (per crystal)
Q-67	–	875	150	60	200	10	5.74(6)	–
Q-85	–	875	150	60	200	10	5.81(16)	–
Q-84	Px	875	150	60	200	10	5.95(19)	–
Q-89	Px	875	150	60	200	10	5.66(5)	–
Q-70	Px	875	150	60	200	10	5.85(27)	1±1
Q-90	Am	875	150	60	200	10	5.48(5)	–
Q-76	Am	875	150	60	200	10	5.86(8)	1±1

^a^Crystal type added: Px = augite, Am = hornblende

^b^Experimental temperature (in °C)

^c^Experimental pressures and times at pressure of first and second steps of experiment

^d^Dissolved H2O contents based on 4–8 analyses. Values in parentheses represent standard errors in terms of least units cited; thus, 5.74 (6) indicates a standard error of 0.06 wt%

**Fig. 1:**
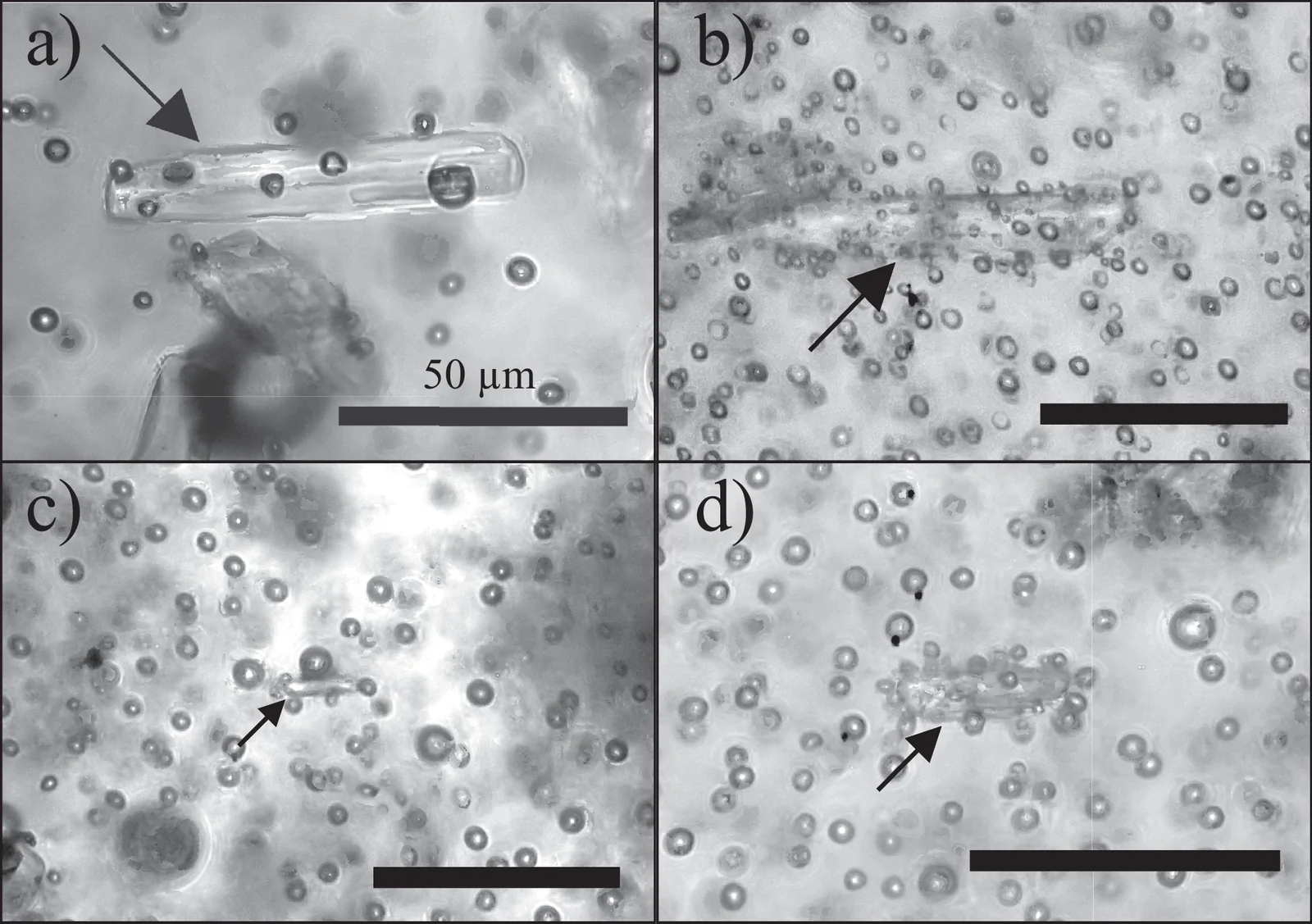
Photomicrographs of bubbles nucleated on augite and hornblende crystals: **a** bubbles on augite at Δp = 20 MPa (Q-71); **b** bubbles on augite at Δp = 45 MPa (Q-64); and **c**, **d** bubbles on hornblende at Δp = 45 MPa (Q-63). All scale bars are 50 µm long

All decompressed samples were analyzed for crystal and bubble numbers and sizes (Table 4). The three dimensions of thirty (30) randomly selected crystals in each sample were measured using the petrographic microscope following the methods of Befus et al (2015), and then, the number of bubbles that were clearly attached to each crystal was counted. Because augite and hornblende are reasonably transparent, bubbles attached to all sides of the crystals could be counted (Fig. 1). Another fifty (50) crystals were randomly selected in each sample that used 20–45 µm crystals, and bubbles were counted on those crystals while noting whether each bubble appeared to nucleate on a flat crystal face or at the corner or edge of the crystal.

**Table 4:** Decompression experiments

Run	Type and size^a^ (µm)	*T*^b^ (°C)	P_2_^c^ (MPa)	*t*_2_^c^(min)	P_F_^c^ (MPa)	*t*_F_^c^ (min)	*N*^d^ (#/crystal)	*N*_*SA*_^e^ (#/µm^2^)
Q-86	Px, 20–45	875	200	10	185	5	1±2	0.0004±0.0005
Q-71	Px, 20–45	875	200	10	175	5	5±4	0.0017±0.0010
Q-64	Px, 20–45	875	200	10	150	5	27±19	0.0110±0.0027
Q-78	Px, <20	875	200	10	155	5	2±1	–
Q-77	Am, 20–45	875	200	10	175	5	6±4	0.0024±0.0013
Q-66	Am, 20–45	875	200	10	170	5	10±8	0.0041±0.0021
Q-63	Am, 20–45	875	200	10	150	5	32±21	0.0109±0.0031
Q-108	Am, <20	875	180	10	175	5	1±1	–

^a^Crystal type (Px = augite, Am = hornblende) and size fraction of crystals

^b^Experimental temperature (in °C)

^c^Experimental pressures and times at pressure of second and final steps of experiment. All experiments initially sintered at 875 °C and 150 MPa for 1 h

^d^Average number of bubbles nucleated on crystals

^e^Average number of bubbles per surface area of crystals

## Results

### Starting conditions

Dissolved H_2_O contents in samples quenched after equilibrating at 200 MPa are, on average, 5.76 ± 0.16 wt% (Table 3). This H_2_O concentration equates to a saturation pressure of ~ 195 MPa, based on the solubility model of Liu et al (2005). We thus assume that all other samples had saturated at 195 MPa before pressure was lowered, except for Q-108, which used part of Q-90 as starting material. Q-90 had 5.48 wt% dissolved H_2_O, equal to a saturation pressure of 180 MPa. Careful examination of the augite and hornblende crystals indicates that no significant change in shapes from either melting or crystallization occurred during the experiments.

Some crystals in the samples quenched at 200 MPa have bubbles attached to them, formed during sintering (Blandon et al. 2023). Thirty crystals in two samples (one containing augite and one containing hornblende) were selected, and the number of these sintering-derived bubbles was counted, and averages 1 ± 1 bubbles per crystal (Table 3). We assume that the same number of sintering-derived bubbles formed in all samples, and thus, for the decompression experiments, we subtracted 1 from the number of bubbles counted on each crystal (unless no bubbles were attached). Given the large number of bubbles on the surface of crystals, we take the resulting error to be small in the subsequent analysis.

Small magnetite crystals (≤ 5 µm in size) grew in the melt during sintering and compression (they were not present after the initial 5-day hydration). Such crystals also formed in similar experiments reported in Aubin and Gardner (2024). Their presence is unavoidable, and we ignore them for this study.

### Decompression experiments

All samples that were decompressed have an outer rind, a few 10 s of microns thick, that contains numerous small bubbles. Such rinds are ubiquitous in experimental decompressions and result from bubbles nucleating at the contact between the sample and metal capsule (e.g., Mourtada-Bonnefoi and Laporte 1999; Mangan and Sisson 2000; Gardner 2009; Aubin and Gardner 2024). We avoided those rinds and focused on crystals located in the interior of the samples (Fig. 1). As seen in Fig. 1, there are bubbles in the interior of all samples that did not nucleate on the augite or hornblende crystals. Some of these bubbles are relicts from the sintering process that did not completely resorb during the compression step. Others nucleated on the small magnetite crystals that formed during the sintering process (Aubin and Gardner 2024). Bubbles readily nucleate on magnetite at small degrees of supersaturation (Hurwitz and Navon 1995; Aubin and Gardner 2024), and thus, their presence is unavoidable. For this study, we ignore all bubbles that did not nucleate on augite or hornblende crystals.

Four decompressions used augite crystals (Table 4). Three used the 20–45 µm crystal-size fraction; those crystals have mutually perpendicular principal axes of average length 39.9(± 22), 15.0(± 7.7), and 10.0(± 4.8) µm (e.g., Fig. 1a, b). The fourth used the < 20 µm sized fraction with lengths 5.4(± 2.0), 2.6(± 0.7), and 2.4(± 0.9) µm. Four decompressions used hornblende crystals (Table 4). Three used the 20–45 µm size fraction of hornblende, with average lengths of 36.8(± 16.4), 16.1(± 7.1), and 11.1(± 4.7) µm (Fig. 1c, d). The fourth used smaller sized hornblende with lengths 14.5(± 7.9), 5.4(± 2.5), and 5.0(± 2.9) µm.

The nearly instantaneous pressure drops generated supersaturation pressures (Δp) of 5 to 45 MPa, equal to the difference between saturation pressure (based on initial H_2_O contents) and final pressure given in Table 4. Regardless of Δp, all bubbles that had nucleated on augite and hornblende are found to be 1–6 µm in diameter (Fig. 1). In samples that used the 20–45 µm size fractions, the proportion of crystals that seeded bubbles increased as Δp increased. At Δp = 5 to 10 MPa, bubbles nucleated on ~50% of the augite and amphibole crystals, whereas bubbles nucleated on 87–93% of crystals at Δp = 20 to 25 MPa, and bubbles nucleated on every crystal at Δp = 40 to 45 MPa.

The number of bubbles nucleated on each crystal increases as Δp increases (e.g., Fig. 1a, b). At Δp = 5 to 10 MPa, 64–94% of augite and hornblende crystals seeded 1–2 bubbles (in excess of those formed during sintering), with up to seven bubbles on a few of the crystals. At Δp = 20 to 25 MPa, an average of 5–10 bubbles nucleated on crystals, with up to 28 on some (Fig. 1a). At Δp = 40 to 45 MPa, 2 to 72 bubbles nucleated on crystals, with more than 60% of them seeding 20 or more (Fig. 1b, d). Interestingly, similar values of Δp result in similar numbers of bubbles on augite and hornblende (Table 4). The locations of 4037 bubbles were analyzed, and 97.5% of them appear on flat surfaces as opposed to being on corners or edges, regardless of Δp or crystal type (Fig. 1).

**Fig. 2:**
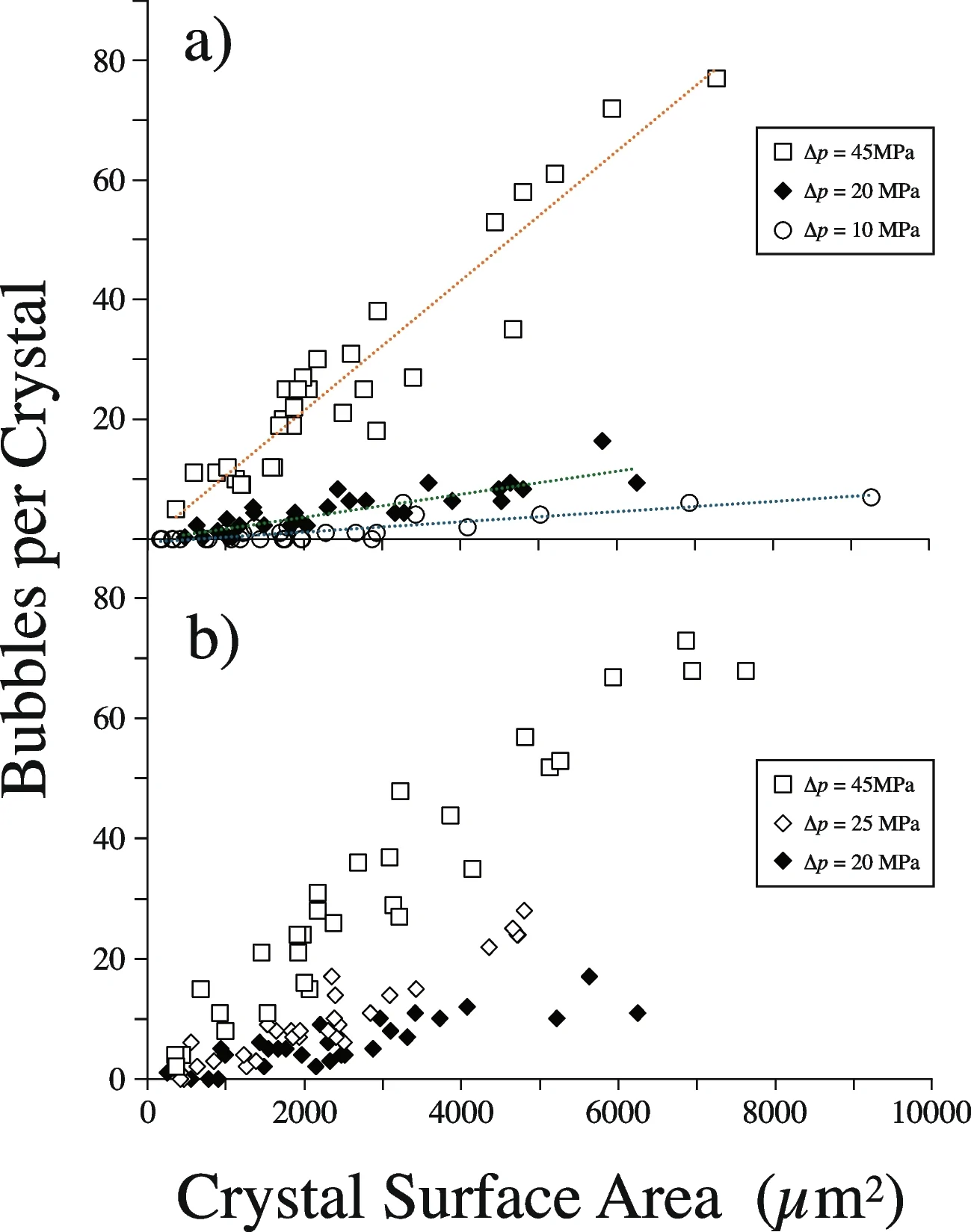
Number of bubbles nucleated on the surface area (µm^2^) of **a** augite crystals or **b** hornblende crystals, at Δp = 10 MPa (open circles), Δp = 20 MPa (solid diamonds), Δp = 25 MPa (open diamonds), and Δp = 45 MPa (open squares)

**Fig. 3:**
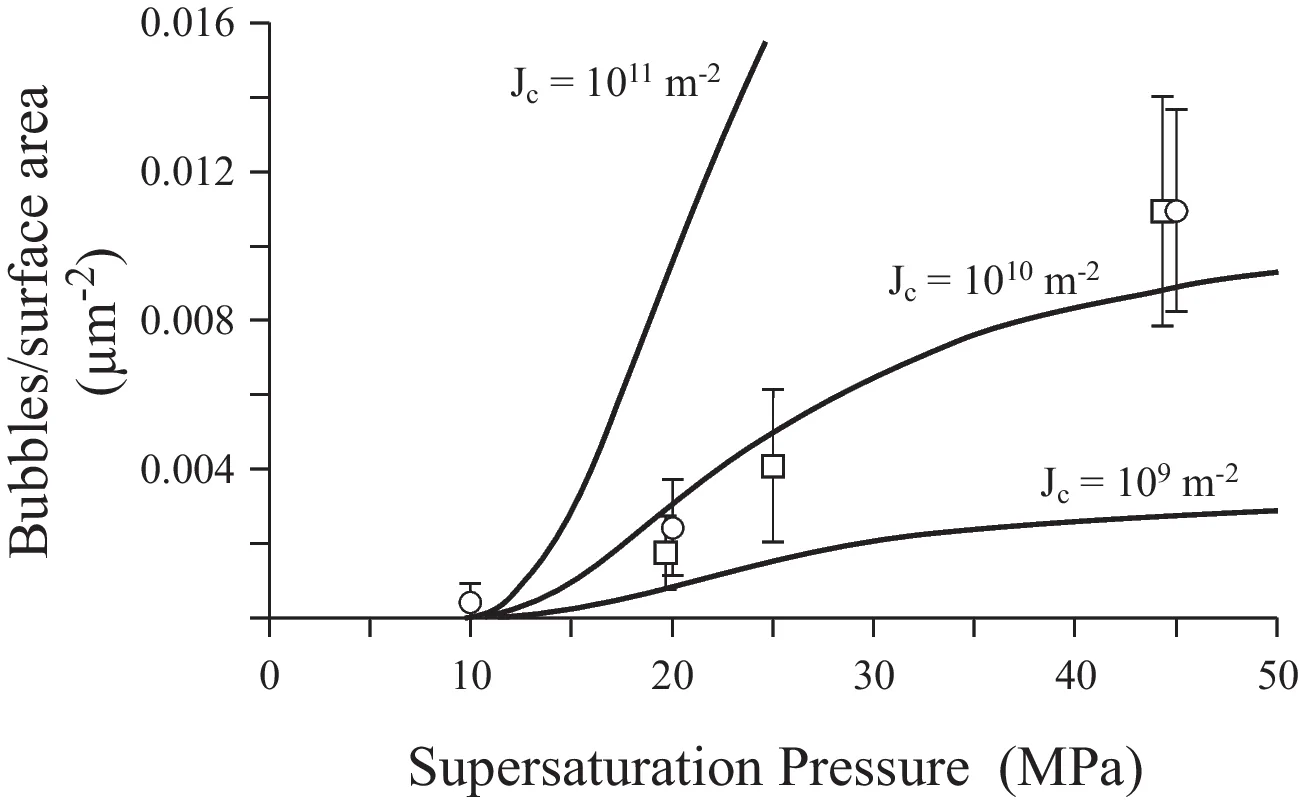
Average number of bubbles per surface area of augite (circles) and hornblende (squares) as functions of supersaturation pressure (Δp). Curves are solutions to Eq. 6 for three different assumed values of ${J}_{c}$ (see “Discussion” for details)

At a given Δp, more bubbles nucleated on larger crystals than on smaller ones (Fig. 1c, d). To investigate this relationship further, we calculated the surface areas of crystals in decompressions that used the 20–45 µm size fraction by assuming all are prismatic cuboids (Fig. 1). We find that augite has surface areas of 167–9248 µm^2^ and hornblende are 254–7629 µm^2^. There is a strong relationship between the number of bubbles on a crystal and the surface area of that crystal at any given Δp (Fig. 2). At similar values of Δp, the number of bubbles per surface area is nearly the same on hornblende and augite crystals. By calculating the number of bubbles per surface area of each crystal, we find that the average number of bubbles per surface area increases with increased Δp (Fig. 3).

## Discussion

Overall, bubble nucleation on augite and hornblende is almost identical at a given Δp in terms of the number of bubbles nucleated, the number of bubbles per crystal surface area, and the proportions of crystals that seeded bubbles. We thus assume in the discussion below that the kinetics of heterogeneous bubble nucleation on augite and hornblende in a rhyolitic melt are the same within experimental uncertainty.

### Efficiency of augite and hornblende for bubble nucleation

Modeling heterogeneous bubbles nucleation requires knowing the value of ${\alpha}_{i}$ (e.g., Eq. 1). It is very difficult to accurately measure the wetting angles of bubble on crystals in our experiments, making it impossible to determine ${\alpha}_{i}$ from the wetting angles of bubble on crystals (e.g., Hurwitz and Navon 1994). To first order, however, ${\alpha}_{i}$ can be approximated from the ratio of the minimum supersaturation pressures required for homogeneous or heterogeneous nucleation in the same melt from the following,

2 $${\alpha}_{i}= {\left(\frac{{\Delta p}_{\mathrm{H}\mathrm{E}\mathrm{T}}}{{\Delta p}_{\mathrm{H}\mathrm{O}\mathrm{M}}}\right)}^{2}$$

where ${\Delta p}_{\mathrm{H}\mathrm{O}\mathrm{M}}$ and ${\Delta p}_{\mathrm{H}\mathrm{E}\mathrm{T}}$ are the minimum supersaturations for homogeneous and heterogeneous nucleation, respectively (Hurwitz and Navon 1994; Hajimirza et al. 2021). In this study, bubbles nucleated on hornblende at Δp = 5 MPa and on augite at 10 MPa, which suggests that ${\Delta p}_{\mathrm{H}\mathrm{E}\mathrm{T}}$ ≤ 5 MPa for both. The high-silica rhyolitic melt used in this study has been used in other studies of homogeneous bubble nucleation (Gardner and Ketcham 2011; Gonnermann and Gardner 2013; Hajimirza et al. 2019). Those found that when the melt contained ~ 5.5–6.0 wt% dissolved H_2_O, bubbles do not nucleate homogeneously at Δp = 100–115 MPa but do at Δp ≥ 125 MPa, indicating that ${\Delta p}_{\mathrm{H}\mathrm{O}\mathrm{M}}$ = 120 ± 5 MPa. From these supersaturation pressures, we estimate that ${\alpha}_{i}$ ≤ 0.0017 ± 0.0002 for both hornblende and augite (Table 5).

**Table 5:** Heterogeneous bubble nucleation in rhyolite melt

Crystal type	$\triangle p_{HET}^a$ (MPa)	α	References^b^
Augite	≤5	0.0017(2)	1
Hornblende	≤5	0.0017(2)	1
Magnetite	<5	<0.002	2,3,4
Hematite (tips)	~20	~0.03	3
Hematite (lengths)	~50	~0.17	3
Plagioclase	–	1	2,3

^a^Minimum supersaturation (in MPa) needed for heterogeneous bubble nucleation.  Bubbles do not nucleate on plagioclase in rhyolite melts

^b^References: 1, this study; 2, Hurwitz and Navon (1994); 3, Gardner and Denis (2004); 4, Aubin and Gardner (2024)

In other studies, bubbles were found to nucleate on magnetite in hydrous rhyolitic melts at Δp ≤ 5 MPa (Hurwitz and Navon 1994; Gardner and Denis 2004; Aubin and Gardner 2024). Augite and hornblende are thus similarly efficient as magnetite for heterogeneous bubble nucleation. In contrast, plagioclase does not seed bubbles in rhyolitic melt (Hurwitz and Navon 1994; Gardner and Denis 2004), and hence, ${\alpha}_{i}$ = 1. In contrast to those results in rhyolitic melts, Plese et al (2018) found that bubbles readily nucleate on plagioclase in andesitic melts, and Larsen (2007) found that Δp must be ~ 50 MPa (${\alpha}_{i}$ ~ 0.17) for bubbles to nucleate on pyroxene in potassic phonolitic melts. More studies of heterogeneous bubble nucleation are thus needed to fully understand the relationship of α with crystal type and melt composition.

Gardner and Denis (2004) found that bubbles nucleated on the tips of hematite rods and along the lengths of those rods at very different supersaturations suggesting that ${\alpha}_{i}$ can vary with crystal structure or the different geometry at the tips versus along the sides (e.g., Schmelzer 1986; Schmelzer and Mahnke 1986; Baidakov and Boltachev 1999; Qian and Ma 2009; Hedges and Whitelam 2012). In this study, bubbles nucleated at very low values of Δp mainly on flat surfaces (97.5% of the bubbles) but also on corners and edges of both amphibole and augite. Thus, if there are any differences in nucleation kinetics with amphibole or augite structure or surface geometry, it must occur at extremely small values of Δp.

### Spacing between nucleation sites on augite and hornblende

The number of bubbles nucleated on small augite and hornblende approaches 1 per crystal as surface area approaches 200 µm^2^, even at Δp =45 MPa (Fig. 2). Aubin and Gardner (2024) also found that at high values of Δp only one bubble nucleated on magnetite crystals that were ~ 1–5 µm in size (surface areas = 6–150 µm^2^). Crystals larger than ~ 200 µm^2^, however, can seed more than one bubble, and the number increases with surface area and higher Δp (Fig. 2). Aubin and Gardner (2024) found that the number of bubbles that nucleated on large magnetite also increased as Δp increased, but because magnetite is opaque, not all sides could be examined, and so, we cannot determine the complete number of bubbles per surface area.

**Fig. 4:**
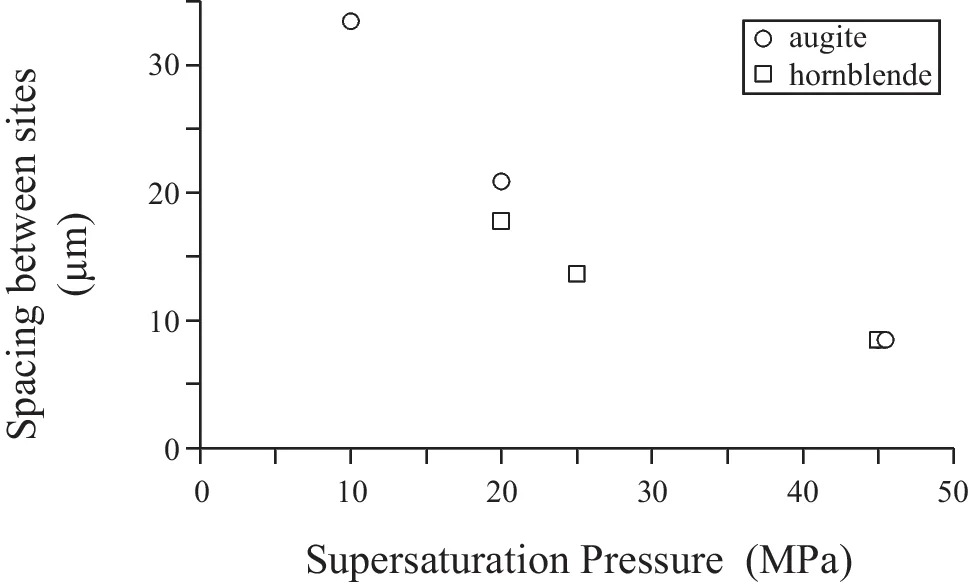
Calculated average spacing between nucleation sites on augite and hornblende crystals as functions of supersaturation pressure (Δp). Spacings were calculated from the average bubbles per surface area assuming that each bubble records a nucleation site activated at the given Δp

The average number of bubbles per surface area increases with increased Δp (Fig. 3), such that at Δp = 10 MPa, there is, on average, 1 bubble per ~2500 µm^2^, but at Δp = 45 MPa, there is ~1 bubble per ~90 µm^2^. If we assume that every bubble represents a nucleation site that was activated by a given value of Δp, then we can calculate the average spacing between sites from the square root of the surface area. We find that the average spacing decreased from ~30 µm at Δp = 10 MPa to ~ 9 µm at Δp = 45 MPa (Fig. 4).

The distance between nucleation sites is thus on average shorter at higher values of Δp (Fig. 4). We interpret this shrinking distance between nucleation sites to reflect the tradeoff between the rate of nucleation and the rate of H_2_O diffusion into bubbles already nucleated. As Δp increases, the free-energy barrier to heterogeneous bubble nucleation decreases rapidly, leading to a strong increase in ${J}_{\mathrm{H}\mathrm{E}\mathrm{T}}$ (Eq. 1). As such, at greater Δp, diffusion of dissolved H_2_O into nearby bubbles is too slow to prevent further nucleation, thus leading to a greater number of bubbles nucleated per unit area.

### Modeling heterogeneous bubble nucleation on crystal populations

Gardner et al (2023) proposed that ${J}_{\mathrm{H}\mathrm{E}\mathrm{T}}$ should be modified to include the number density of sites available for bubbles to nucleate (i.e., the ${\beta}_{i}$ term in Eq. 1). That correction assumed that there is one nucleation site per crystal, which our experiments show holds only for crystals smaller than ~ 5 µm. On larger crystals, the number of nucleation sites increases with surface area and depends on Δp (Figs. 2 and 3). We now use these results to reformulate ${J}_{\mathrm{H}\mathrm{E}\mathrm{T}}$ to include that dependence on crystal surface area.

Given that we are considering nucleation as a function of surface area, we first consider a population of nucleated bubbles in terms of area density $N^{\prime}$ (instead of volume density N). The characteristic time taken for a bubble to nucleate on a given surface area can be predicted as

3 $${t}_{\mathrm{n}\mathrm{u}\mathrm{c}}=\frac{1}{{J}_{\mathrm{H}\mathrm{E}\mathrm{T}}^{\prime}{A}_{\mathrm{n}\mathrm{u}\mathrm{c}}}$$

where ${J}_{\mathrm{H}\mathrm{E}\mathrm{T}}^{\prime}$ is the nucleation rate per unit area per unit time, and ${A}_{\mathrm{n}\mathrm{u}\mathrm{c}}$ is the area of the crystal per bubble, which is the inverse of the area density of bubbles: ${A}_{\mathrm{n}\mathrm{u}\mathrm{c}}=1/N^{\prime}$. Expressing nucleation rates on a per unit area basis is consistent with theoretical treatments of heterogeneous nucleation processes (Blander and Katz 1975), and it is straightforward to convert between ${J}_{\mathrm{H}\mathrm{E}\mathrm{T}}^{\prime}$ (per area, Eq. 3) and ${J}_{\mathrm{H}\mathrm{E}\mathrm{T}}$ (per volume, Eq. 1), as we will show later.

As we note above, observations of very small crystals show that the surface area required to nucleate a single bubble is much smaller than the value of ${A}_{\mathrm{n}\mathrm{u}\mathrm{c}}$ measured from larger crystals that host multiple bubbles. This implies that nucleation rate is not controlled exclusively by the availability of nucleation sites on the crystal surface—some other process is limiting nucleation on larger crystals. We infer that, once a bubble nucleates on a crystal surface, diffusion of H_2_O into the bubble reduces supersaturation locally and suppresses the formation of new bubbles within an area that grows with time. This defines a characteristic diffusion timescale.

4 $${t}_{\mathrm{d}\mathrm{i}\mathrm{f}\mathrm{f}}=\frac{{A}_{\mathrm{n}\mathrm{u}\mathrm{c}}}{D}$$

where D is the diffusivity of H_2_O. This is the characteristic time for diffusion into a bubble to deplete H_2_O from an area of crystal surface around it of crystal-size ${A}_{\mathrm{n}\mathrm{u}\mathrm{c}}$. We propose that the area density of nucleation events on a crystal is controlled by the balance between the characteristic time to nucleate a particular number of bubbles per unit area ${t}_{\mathrm{n}\mathrm{u}\mathrm{c}}$, and the characteristic time for diffusion to inhibit bubble nucleation ${t}_{\mathrm{d}\mathrm{i}\mathrm{f}\mathrm{f}}$. Note that this definition of ${t}_{\mathrm{d}\mathrm{i}\mathrm{f}\mathrm{f}}$ differs from that used in Toramaru and Miwa (2008), who measure the diffusive timescale associated with the growth of bubbles whereas we present the timescale describing the growth of the diffusive exsolution halo, inside which nucleation is suppressed. No more nucleation will occur when the entire crystal surface has been covered by these exclusion haloes. For a given area density of bubbles, we now have two measures of when nucleation will be completed. By setting ${t}_{\mathrm{n}\mathrm{u}\mathrm{c}}={t}_{\mathrm{d}\mathrm{i}\mathrm{f}\mathrm{f}}$, we thus obtain the expression

5 $${N}^{\prime}=\sqrt{\frac{{J}_{\mathrm{H}\mathrm{E}\mathrm{T}}^{\prime}}{D}}$$

which gives the nucleation area density in terms of the per unit area nucleation rate and diffusivity.

We can rewrite the formulation for ${J}_{\mathrm{H}\mathrm{E}\mathrm{T}}^{\prime}$ to give

6 $${J}^{\prime}_{\mathrm{H}\mathrm{E}\mathrm{T}}={J}^{\prime}_{c} \mathrm{e}\mathrm{x}\mathrm{p}\left(-\frac{16\pi {\sigma }^{3}}{3{k}_{B}T\Delta {p}^{2}}{\alpha}_{i}\right)$$

where ${J}_{c}^{\prime}$ is the pre-exponential factor. This formulation is similar to the formulation of ${J}_{\mathrm{H}\mathrm{E}\mathrm{T}}$ in Eq. 1, except that it is on a per-area basis rather than a per-volume basis, and it does not include the term ${\beta}_{i}$ (the effects that ${\beta}_{i}$ encoded will be discussed below). This definition of ${J}_{\mathrm{H}\mathrm{E}\mathrm{T}}{\prime}$ leads to an expression for the nucleation density on a per-area basis.

7 $${N}^{\prime}= \sqrt{\frac{{J}_{c}^{\prime}}{D}\mathrm{e}\mathrm{x}\mathrm{p}\left(-\frac{16\pi {\sigma }^{3}}{3{k}_{B}T\Delta {p}^{2}}{\alpha}_{i}\right)}$$

Importantly, because we can calculate the appropriate values for diffusivity ($D={10}^{-10.6}{ \mathrm{m}}^{2} {\mathrm{s}}^{-1}$ for rhyolite melt with 5 wt% H_2_O; Zhang et al. 2010) and surface tension given by the Tolman-corrected function $\sigma (\Delta p)$ from Hajimirza et al. (2019), and we have determined α = 0.0017 from our experiments, this leaves ${J}_{c}^{\prime}$ as a fit parameter to compare solutions to Eq. 7 to our experimental dataset (Fig. 3). We find that a value of ${J}_{c}^{\prime}\approx {10}^{10} {\mathrm{m}}^{-2}$ matches the experimental results well, with the caveat that this holds only for the experimental conditions.

In the formulation above, $N^{\prime}$ is the number of bubbles per area of crystal. As shown in Fig. 2, the number of bubbles increases approximately linearly with crystal area; this linearity does not hold for very small crystals, however, which tend to host a single bubble even when the total surface area of a crystal (A) is much smaller than ${A}_{\mathrm{n}\mathrm{u}\mathrm{c}}$. This is readily explained within the diffusion-limited mechanism that we propose for larger crystals. Self-evidently, the diffusive shut-off mechanism can only apply after at least one bubble has formed on the surface of a crystal. Therefore, the first bubble to form on any crystal (which is the only bubble to form on crystals with $A\ll {A}_{\mathrm{n}\mathrm{u}\mathrm{c}}$) is not subject to the diffusion limit; this manifests as a higher apparent $N^{\prime}$ for these small crystals. In order to capture the transition in regimes from one bubble per crystal to multiple bubbles per crystal, we introduce a correction factor χ to give ${N}_{\mathrm{c}\mathrm{o}\mathrm{r}\mathrm{r}}^{\prime}=\sqrt{{J}_{\mathrm{H}\mathrm{E}\mathrm{T}}^{\prime}\chi /D}$, where χ is given by

8 $$\chi =1+ {\left(\frac{{A}_{\mathrm{n}\mathrm{u}\mathrm{c}}}{A}\right)}^{2}$$

This parameter is a correction to the predicted bubble number density to capture the transition from one bubble per crystal regime at low A to multiple bubbles per crystal regime at high A. This allows us to formulate an expression for the expected number of bubbles on a crystal N as a function of the crystal surface area A. The increase in χ at low A ensures that at least one bubble will form on every crystal. Noting that ${A}_{\mathrm{n}\mathrm{u}\mathrm{c}}=\sqrt{D/{J}_{\mathrm{H}\mathrm{E}\mathrm{T}}^{\prime}}$ (from setting ${t}_{\mathrm{n}\mathrm{u}\mathrm{c}}={t}_{\mathrm{d}\mathrm{i}\mathrm{f}\mathrm{f}}$ in Eqs. 3 and 4), and that $N={N}^{\prime}A$, we find

9 $$\begin{array}{l}N=\sqrt{1+\frac{A^2}{A_\mathrm{nuc}^2}}=\sqrt{1+A^2\frac{J_\mathrm{HET}'}D}\\=\sqrt{1+\frac{A^2J_c'}D\mathrm{exp}\left(-\frac{16\pi\sigma^3}{3k_BT\Delta p^2}\alpha_i\right)}\end{array}$$

**Fig. 5:**
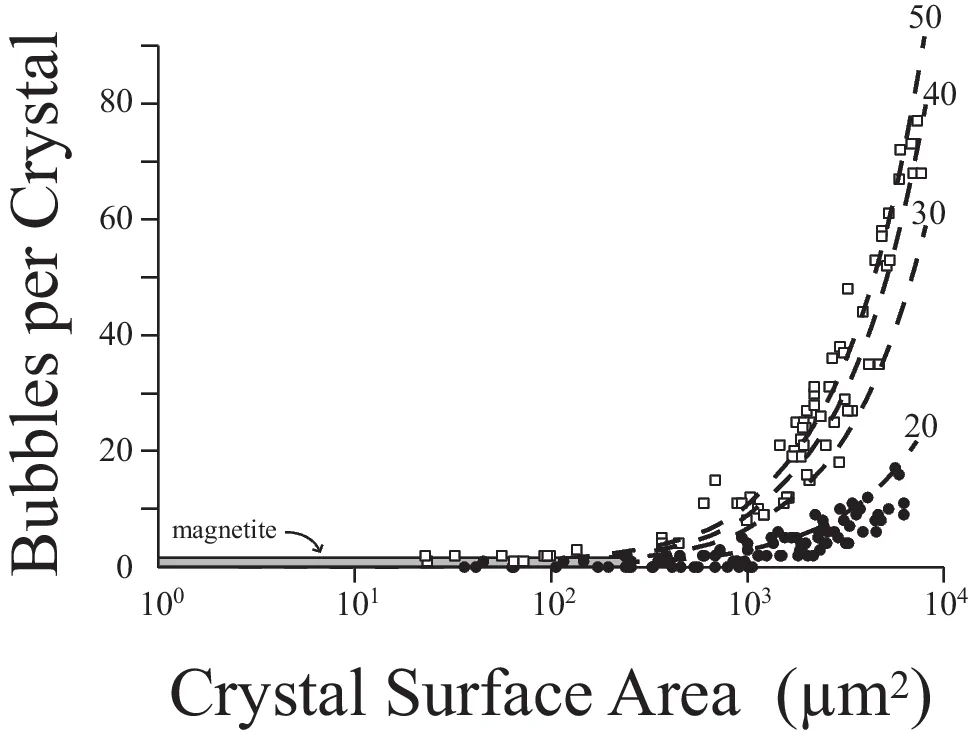
Numbers of bubbles nucleated on crystals as functions of crystal-size surface area (µm^2^). Dashed curves are solutions of Eq. 8 for values of Δp = 20, 30, 40, and 50 MPa (see text for discussion). Experimental results are shown for bubbles on augite and hornblende at Δp = 20–25 MPa (solid circles) and at Δp = 45 MPa (open squares). Also shown are bubble numbers on 1–5 µm magnetite (surface areas = 1–150 µm^2^) from Aubin and Gardner (2024)

In the limit of $A\ll {A}_{\mathrm{n}\mathrm{u}\mathrm{c}}$, this reduces to N=1, and in the limit of $A\gg {A}_{\mathrm{n}\mathrm{u}\mathrm{c}}$, this reduces to $N={N}^{\prime}A$, as required. We note that this approach captures the effects that Gardner et al (2023) originally cast as the parameter ${\beta}_{i}$ (see Eq. 1). By using our previously determined value for ${J}_{c}^{\prime}$ (${10}^{10}{\mathrm{m}}^{-2}$), we can use Eq. 9 to forward model the number of bubbles per crystal area (Fig. 5). Solutions for different values of Δp match the experimental data well.

So far, we have determined heterogeneous nucleation in terms of area density. From this, we can determine the volumetric number density of bubbles, ${N}_{b}$, nucleated in magma containing crystals in number density ${{N}_{c}}$. For monodisperse crystals with identical surface area A, we have

10 $${N}_{b}= {N}_{c}A\sqrt{\chi \frac{{J}_{\mathrm{H}\mathrm{E}\mathrm{T}}^{\prime}}{D}}$$

More generally, for crystals of different sizes, we have

11 $${N}_{b}= {\int}_{0}^{\infty }F\left(A\right)\sqrt{\chi \frac{{J}_{\mathrm{H}\mathrm{E}\mathrm{T}}^{\prime}}{D}}dA$$

where F(A) is the crystal surface area distribution (i.e., the number of crystals with area A per unit volume in units of m^−3^) such that ${N}_{c}={\int}_{0}^{\infty }F\left(A\right)dA$. Therefore, $F\left(A\right)$ can be explicitly found from a crystal-size distribution. Note that in our analysis and model, crystal-size distribution and bubble number distribution refer to sizes or numbers on a per-volume basis (with units of m^−3^) and should not be confused with population density distributions (m^−4^).

The number of bubbles nucleated per unit volume in the system can be calculated from the supersaturation pressure and crystal-size distribution from either Eqs. 12 or 11, depending on the size distribution of crystals. Using the characteristic nucleation time from Eq. 3, we can calculate ${J}_{\mathrm{H}\mathrm{E}\mathrm{T}}$ (for an individual crystal type) by comparing the number of bubbles that nucleate on the crystal with the nucleation time and volume of melt surrounding the crystal. Here, it becomes more convenient to express the nucleation time as ${t}_{\mathrm{n}\mathrm{u}\mathrm{c}}=1/\sqrt{{J}_{\mathrm{H}\mathrm{E}\mathrm{T}}^{\prime}D}$ by eliminating ${A}_{\mathrm{n}\mathrm{u}\mathrm{c}}$ from Eqs. 3 and 4, which results in ${J}_{\mathrm{H}\mathrm{E}\mathrm{T}}$ being given by

12 $${J}_{\mathrm{H}\mathrm{E}\mathrm{T}}= \frac{{N}_{b}}{{t}_{\mathrm{n}\mathrm{u}\mathrm{c}}}=A{N}_{c}\sqrt{\chi \frac{{J}_{\mathrm{H}\mathrm{E}\mathrm{T}}^{\prime}}{D}}\sqrt{{J}_{\mathrm{H}\mathrm{E}\mathrm{T}}^{\prime}D}=A{N}_{c}\sqrt{\chi }{J}_{\mathrm{H}\mathrm{E}\mathrm{T}}^{\prime}$$

As with Eq. 11, integrating over the crystal population allows the nucleation rate for the entire system to be retrieved, giving

13 $${J}_{\mathrm{H}\mathrm{E}\mathrm{T}}= {\int}_{0}^{\infty }F\left(A\right)\sqrt{\chi }{J}_{c}^{\prime}\mathrm{e}\mathrm{x}\mathrm{p}\left(-\frac{16\pi {\sigma }^{3}}{3{k}_{B}T\Delta {p}^{2}}{\alpha}_{i}\right)dA$$

which takes the form of the heterogeneous nucleation rate equation in Eq. 1, allowing us to identify the relationships between our per unit area analysis of nucleation on large crystals and the volumetric nucleation rate. At present, this approach assumes that there is nucleation on every crystal. Our study demonstrates that in practice, diffusion of volatiles between crystals will reduce this effective rate at sufficiently low values of Δp.

### Calculation of bubble number density for a known crystal population

We provide a spreadsheet that allows users to enter crystalsize data and known parameters (temperature, pressures, dissolved H_2_O contents) to estimate both the number density of bubbles nucleated on each crystal-size and the total number density of bubbles (see Supplemental Information). The number of crystals of each size class per unit volume can be calculated from a known distribution of crystals using the population density distribution of sizes ${n}^{\prime}$ (not to be equated with ${N}_{c}$ used earlier). Here, ${n}^{\prime}=l C\mathrm{exp}(ml)$, where *l* is the crystal length and *m* and *C* are constants that capture the slope and intercept of the population distribution. $F\left(A\right)$ is then computed as the area of crystal per volume of melt as $F\left(A\right)={n}^{\prime}A$, where area is calculated. In the spreadsheet, we assume crystals are cubic shaped.

**Fig. 6:**
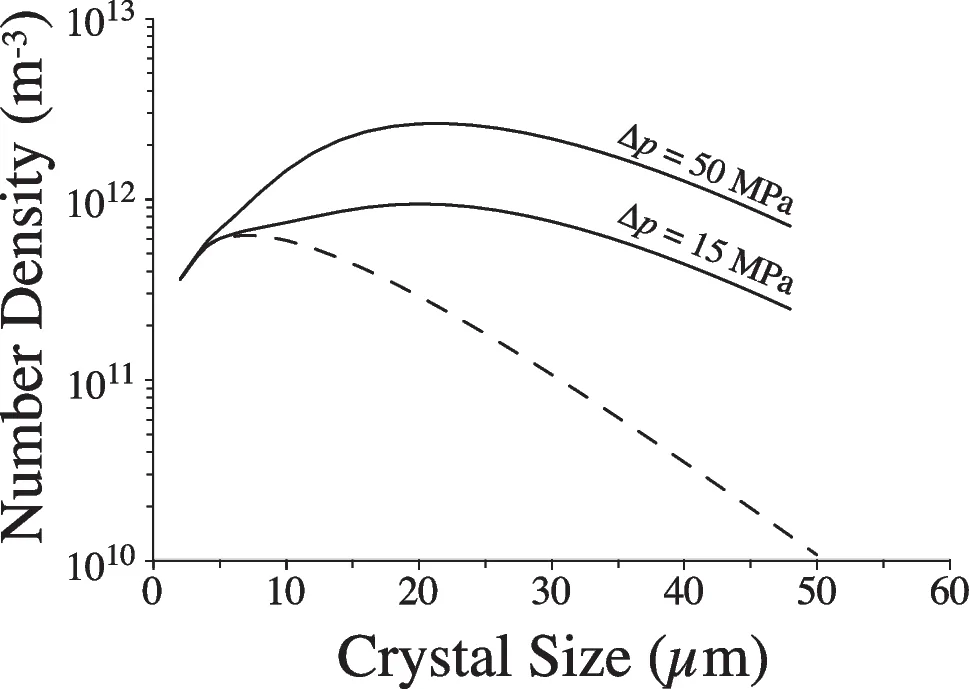
Number densities of bubbles expected to nucleate at Δp = 15 or 50 MPa on crystals of different sizes in a population given by the dashed curve, calculated from Eq. 12. The total number density of bubbles nucleated is 10^13.2^ m^−3^ and 10^13.6^ m^−3^ at Δ*p* = 15 MPa and 50 MPa, respectively

As an example, we use the crystal-size distribution of clinopyroxene microlites generated in decompression experiments using Inyo pyroclastic rhyolite (Castro and Gardner 2008). For our purposes, we extend the size range to 50 µm but maintain the overall distribution which is skewed toward greater numbers of small crystals (Fig. 6). We assume an initial H_2_O content of 5 wt%, *T* = 875 °C, and α = 0.0017. We calculated bubble number density distributions for two different supersaturations (Fig. 6). As expected, the number densities of bubbles nucleated (i.e., bubbles per unit volume of magma) on crystals smaller than ~ 5 µm equal those of the crystals, but on larger crystals, bubble number densities far exceed crystal number densities. The excess of number of bubbles on larger crystals increases with crystal size (area) and Δ*p*. The peak in bubble number densities occurs on ~20–25 µm crystals, but that results from the crystal-size distribution being skewed toward small sizes. Overall, the total number density of bubbles nucleated at Δ*p* = 15 MPa is 10^13.2^ m^−3^ and 10^13.6^ m^−3^ at Δ*p* = 50 MPa.

In our examples, ${N}_{b}$ exceeds ${N}_{c}$ by a factor of three at Δ*p* = 15 MPa and a factor of seven at Δ*p* = 50 MPa. This illustrates that the number density of heterogeneously nucleated bubbles depends strongly on the crystal population because larger crystals provide greater numbers of nucleation sites. Indeed, in our example, the number density of bubbles on 40-µm crystals equals the number density on 8-µm crystals, despite there being about ten times more smaller crystals.

We caution that our model is built by assuming that nucleation occurs on every crystal. In practice, this is reasonable for magnetite, augite, and hornblende at values of Δ*p* ≥ 15–20 MPa (Aubin and Gardner 2024; this study). Any prediction of ${N}_{b}$ at lower values of Δ*p* would likely be overestimated. Our examples also assume α = 0.0017, which is reasonable for magnetite, augite, and hornblende. Predicted values of ${N}_{b}$ will likely differ for other crystal types. Finally, our calculations assumed ${J}_{c}={10}^{10} {\mathrm{m}}^{-2}$, which is valid for augite and hornblende over the range of Δ*p* investigated (Fig. 3). Regardless of such caveats, our modeling shows that heterogeneous bubble nucleation depends on the full characteristics of the crystal population and not merely on whether a particular mineral type exists.

### Conclusions

Decompression experiments were carried out using hydrous rhyolitic melts seeded with populations of augite or hornblende crystals. The number of bubbles that nucleated on crystals depended strongly on the degree of H_2_O supersaturation in the melt generated by the decompression (Δ*p*) and on the surface area of the crystals present. Crystals larger than ~ 5 µm (those with more surface area) allow more bubbles to nucleate, with the average number of bubbles per surface area increasing with Δ*p*. Smaller crystals seed only one bubble, regardless of Δ*p*. The number of nucleation sites on large crystals increases with Δ*p* because the rate of heterogeneous nucleation increases faster than the rate of H_2_O diffusion into bubbles already nucleated. There are no significant differences in the behavior of bubbles nucleating on augite and hornblende, and the efficiency of heterogeneous nucleation on those crystals equals that found on magnetite by previous studies. These results enabled us to reformulate the equation for calculating the rate of heterogeneous bubble nucleation to include not only the heterogeneous adjustment factor ${\alpha}_{i}$ but also a modified pre-exponential factor that accounts for the characteristics of the crystal population on which the bubbles nucleate. Future studies can expand this analysis for other crystal types and melt compositions.

## Supplementary information

Below is the link to the electronic supplementary material.

ESM 1 (XLSX 31.6 KB)

## Data Availability

All data supporting the findings of this study are available within the paper and its Supplementary Information.  All experimental products are available from the corresponding author upon request.
